# Orexin/Hypocretin Modulation of Cholinergic Signaling within the Prefrontal Cortex in Response to an Immune Challenge

**DOI:** 10.1002/alz70855_099474

**Published:** 2025-12-23

**Authors:** Marla A. Frick, Jennifer L. Woodruff, Yair M. Caudillo, Christian D. Wohlfeld, Claudia A. Grillo, Lawrence P. Reagan, Jim R. Fadel

**Affiliations:** ^1^ University of South Carolina School of Medicine, Columbia, SC, USA; ^2^ WJB Dorn VA Medical Center, Columbia, SC, USA

## Abstract

**Background:**

The basal forebrain cholinergic hypothesis (BFCS) describes the degeneration of cholinergic neurons in Alzheimer's Disease (AD) that is correlated with disease pathology and severity [1]. There has been limited progress in understanding of the vulnerability of this system, suggesting that there are other factors at play, such as altered afferent regulation. We have previously shown that the orexin/hypocretin system modulates cholinergic signaling and neuroinflammation in key regions implicated in AD, such as the BF, hippocampus, and prefrontal cortex (PFC) [2, 3]. Therefore, we hypothesize that orexin downregulation will impair cholinergic response in the PFC following an immune challenge.

**Method:**

Young female rats received bilateral LH injections of control lentivirus or lentiviral‐preproorexin antisense and a microdialysis guide cannula was placed into the PFC. Three weeks later, animals received a 1mg/kg intraperitoneal injection of lipopolysaccharide to induce an immune response. In‐vivo microdialysis with a food‐paired stimulus was performed and brain dialysates were measured for acetylcholine using high‐performance liquid chromatography.

**Result:**

We hypothesize that cholinergic response in the PFC of control females will be significantly increased following a food‐paired stimulus, as we have previously shown in the insular cortex. This effect will be disrupted following orexin downregulation and LPS injection when compared to control females.

**Conclusion:**

Orexin modulates neuroinflammation and cholinergic function in the PFC of female rats. Specifically, downregulation of orexin impairs the appropriate neuroimmune and neurochemical responses in the PFC to an immune challenge. Thus, the well‐documented age‐ and AD‐associated reduction of orexin signaling may be linked to BFCS degeneration via neuroinflammatory mechanisms.

Hampel, H., et al., *Revisiting the Cholinergic Hypothesis in Alzheimer's Disease: Emerging Evidence from Translational and Clinical Research*. J Prev Alzheimers Dis, 2019. **6**(1): p. 2‐15.

Hagar, J.M., et al., *Upregulation of orexin/hypocretin expression in aged rats: Effects on feeding latency and neurotransmission in the insular cortex*. Neuroscience, 2017. **350**: p. 124‐132.

Calva, C.B., H. Fayyaz, and J.R. Fadel, *Increased acetylcholine and glutamate efflux in the prefrontal cortex following intranasal orexin‐A (hypocretin‐1)*. J Neurochem, 2018. **145**(3): p. 232‐244.

